# Review of the pharmacokinetics of French maritime pine bark extract (Pycnogenol^®^) in humans

**DOI:** 10.3389/fnut.2024.1389422

**Published:** 2024-05-02

**Authors:** Jasmin Bayer, Petra Högger

**Affiliations:** Institut für Pharmazie und Lebensmittelchemie, Universität Würzburg, Würzburg, Germany

**Keywords:** Pycnogenol^®^, procyanidins, bioavailability, distribution, metabolism, elimination, pharmacokinetics

## Abstract

The French maritime pine bark extract Pycnogenol^®^ is a proprietary product from *Pinus pinaster* Aiton. It complies with the quality specifications in the *United States Pharmacopeia* monograph “Pine extract” in the section of dietary supplements. Pycnogenol^®^ is standardized to contain 65–75% procyanidins which are a variety of biopolymers consisting of catechin and epicatechin monomeric units. The effects of Pycnogenol^®^ have been researched in a multitude of human studies. The basis for any *in vivo* activity is the bioavailability of constituents and metabolites of the extract. General principles of compound absorption, distribution, metabolism and elimination as well as specific data from studies with Pycnogenol^®^ are summarized and discussed in this review. Based on plasma concentration profiles it can be concluded that low molecular weight constituents of the extract, such as catechin, caffeic and ferulic acid, taxifolin are readily absorbed from the small intestine into systemic circulation. Procyanidin oligomers and polymers are subjected to gut microbial degradation in the large intestine yielding small bioavailable metabolites such as 5-(3′,4′-dihydroxyphenyl)-γ-valerolactone. After intake of Pycnogenol^®^, constituents and metabolites have been also detected in blood cells, synovial fluid and saliva indicating a substantial distribution in compartments other than serum. In studies simultaneously investigating concentrations in different specimen, a preferential distribution of individual compounds has been observed, e.g., of ferulic acid and 5-(3′,4′-dihydroxyphenyl)-γ-valerolactone into synovial fluid compared to serum. The main route of elimination of constituents and metabolites of the French pine bark extract is the renal excretion. The broad knowledge accumulated regarding the pharmacokinetics of compounds and metabolites of Pycnogenol^®^ constitute a rational basis for effects characterized on a cellular level and observed in human clinical studies.

## 1 Introduction

Among conifers, the pine (*Pinus* L.) is a genus of more than 100 different species which grow widespread in Europe and the Mediterranean basin, Asia and in Northern America. Pine seeds, needles, cones and bark contain different categories of phytochemicals which are essential for plant growth and development as well as having protective functions for the pines (1). Botanical extracts from pine bark have a long-standing history of use in traditional medicine, e.g., for wound healing (2). Nowadays, they are still used as dietary supplements and insights into the scientific basis of their effects is yet increasing. Extracts can be sourced from different *Pinus* (*P*.) species such as *P. densiflora*, *P. maritima*, *P. massonia*, *P. nigra*, *P. pinaster*, *P. radiata*, or *P. sylvestris*. The bark extracts are concentrated sources of phenolic compounds such as procyanidins, flavonoids or phenolic acids. Their content and composition depend on the pine species and the precise extraction solvent and procedure. Therefore, the extract compositions are marked by different concentrations and types of phenolic compounds which account for individual bioactivities (1, 3–7).

Among pine bark extracts, the proprietary French maritime pine bark extract Pycnogenol^®^ is most extensively researched (8). It is produced in a patented procedure from the bark of *P. pinaster* and has a standardized composition (see section “2.2 Composition”). A consistent extract composition facilitates research of its pharmacology with pharmacokinetics describing compound concentrations at specific body sites and pharmacodynamics elucidating bioactivities on a cellular or clinical level. So far, roughly 160 clinical studies with over 10,000 participants have been reported for Pycnogenol^®^. The results suggested a beneficial impact in various chronic disease states, particularly when inflammation and/or oxidative stress contributed to the pathophysiology (9, 10). Documented clinical effects prompted deeper investigations of underlying cellular mechanisms and identification of relevant bioactive molecules. In this context the catechin-derived gut microbial metabolite 5-(3′,4′-dihydroxyphenyl)-γ-valerolactone gained particular interest. This bioactive molecule originates from catechin or epicatechin units which in turn constitute procyanidins that are highly abundant in the French maritime pine bark extract Pycnogenol^®^ (see sections “1.2.2 Pharmacodynamics,” “4.1 Absorption into plasma/serum and concentration profiles,” “5 Biodistribution,” and “6.1 Pre-systemic metabolism: role of gut microbiota”).

### 1.1 Pharmacokinetics govern pharmacodynamics

**FIGURE 1:**
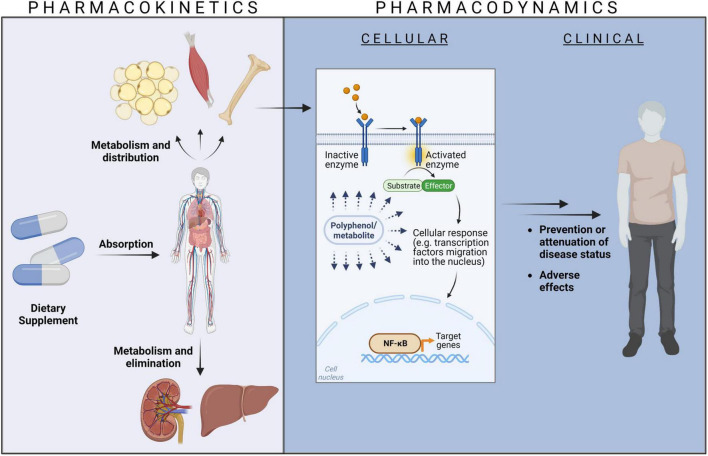
The relationship between pharmacokinetics and pharmacodynamics. Created with BioRender.com.

Dietary phenolic compounds are no drugs and therefore, the term “pharmaco”-kinetics is strictly speaking not precise, but frequently used in the context of natural plant-derived compounds. Alternative phrases such as “biokinetics” (11) or “nutrikinetics” (12) have been created. Though being accurate, these expressions are less prevalent so far. In the body, dietary phenolic compounds are subjected to the same processes as drugs, that is, they have to be taken up *in vivo* (absorption), they are distributed in tissues and organs, they are typically metabolized and finally eliminated (Figure 1). To keep this analogy in mind, the term pharmacokinetics is used in this review.

Sufficiently high concentrations of dietary phenolic compounds at their site of action in tissues and organs trigger cellular events, e.g., at receptors, ion channels, transporter proteins, enzymes, or modulation of DNA transcription. These cellular events might translate into clinical responses like prevention or attenuation of disease states or adverse effects (Figure 1). The bioactivity aspects of dietary phenolic compounds can be described as pharmacodynamics (13), again to remind of the similarity to drug actions.

### 1.2 Methodological approaches

For determination of the pharmacokinetics and pharmacodynamics of dietary extracts, different experimental approaches, yielding specific information, can be chosen (Table 1). Generally, plant-derived extracts pose an interesting challenge to research as they typically comprise a variety of structurally similar and/or heterogeneous constituents which readily undergo metabolic changes. Therefore, in contrast to investigation of single drug molecules, manifold compounds must be monitored and characterized in a “multiplex” proceeding.

**TABLE 1:** Methodological approaches to dietary extract research and examples of information derived from particular experimental settings.

	Pharmacokinetics	Pharmacodynamics
*In vitro*	Solubility and membrane permeability, characterization of the role of transporters Determination of the interaction with specific structures, e.g., plasma proteins Determination of the metabolism in particular cells	Detailed determination of diverse cellular events (caution: stability)
*Ex vivo*	–	Particular approach which allows the investigation of cellular events with authentic serum samples obtained after the intake of the herbal extract, thus containing all bioavailable constituents and metabolites at physiological concentrations. These serum samples are used in cell culture experiments
*In vivo* (human)	Determination of all typical PK parameters (except for the absolute bioavailability)	Detailed determination of the physiological / clinical response in clinical studies: systemic and local (e.g., skin)

#### 1.2.1 Pharmacokinetics

Single pharmacokinetic aspects with potential relevance for compound absorption, distribution, metabolism, or elimination can be studied in detail in cell cultures or other *in vitro* settings. Essential requirements for molecule absorption include solubility and solute membrane permeability. Besides passive diffusion across bilayer membranes, uptake or efflux via membrane transporter molecules is of high significance for compound absorption, distribution and elimination (14–16). Individual plant-derived polyphenols have been identified as transporter substrates (17, 18). Likewise, a Pycnogenol^®^ metabolite was observed to be subjected to facilitated uptake via GLUT-1 transporters into erythrocytes and endothelial cells (19, 20).

For Pycnogenol^®^ constituents and metabolites, interactions with some specific structures, such as matrix proteins (21), erythrocyte membranes (22) and human albumin (23) were characterized *in vitro*. These interactions have implications for pharmacokinetic distribution aspects.

Typically, dietary phenolic substances are extensively metabolized, e.g., by gut bacteria as well as by phase I and phase II reactions in enterocytes and hepatocytes (24). Particularly phase I reactions catalyzed by cytochrome P 450 enzymes (CYP enzymes) have been widely studied in liver microsomes (25) and with recombinant individual CYP enzyme isotypes (26). Phase II reactions typically yield conjugates with e.g., sulfate or glucuronic acid (27). Besides metabolism in gut and liver cells, metabolic transformations can occur in other cells as well. In this context, degradation and conjugation reactions of a Pycnogenol^®^ metabolite have been investigated in erythrocytes employing a metabolomic approach (28).

While *in vitro* experiments yield detailed information on single aspects, *in vivo* studies with human volunteers or patients allow for the determination of typical pharmacokinetic parameters, such as maximum concentrations in serum or plasma (c_*max*_), time of peak concentrations (t_*max*_), area under the curve (AUC), half-live (t½) and other elimination characteristics. Parameters can be determined for all single constituents or metabolites of complex extracts. However, one pharmacokinetic aspect usually cannot be determined in humans, namely the precise absolute bioavailability. Bioavailability describes the rate and extent of compound uptake into systemic circulation (24). Since the constituents of dietary extracts are not completely absorbed from the gastrointestinal tract, the absolute bioavailability specifies the percentage of compounds that are taken up after oral ingestion. The calculation of the absolute bioavailability requires the comparison of the rate and extent of compound uptake after oral and intravenous administration. Intravenous administration might be possible and ethical for single phenolic compounds (29), but not for whole extracts. For the French maritime pine bark extract Pycnogenol^®^, pharmacokinetics were determined in several human studies describing urinary metabolites (30, 31) as well as compounds and metabolites detected in plasma or serum samples after intake of single and multiple doses (32–34).

#### 1.2.2 Pharmacodynamics

Single pharmacodynamic aspects, allowing detailed determination of specific cellular responses, can be studied in detail in cell cultures. Thereby, it is possible to uncover activities of extract constituents at particular cellular signal transduction pathways after interacting with target molecules, e.g., receptors or enzymes. Besides classical antioxidant effects, dietary phenolic compounds or metabolites thereof have been discussed as modulators of DNA transcription based on interaction with transcription factors such as Nf-κB (35, 36) or ligand-dependent transcription factors like the aryl hydrocarbon receptor (37) or peroxisome proliferator-activated receptors (38). They also interact with various signaling-relevant kinases or inhibit specific enzymes such as α-glucosidase or matrix metalloproteinases (MMP). For the French maritime pine bark extract Pycnogenol^®^, cellular effects are mainly related to its anti-inflammatory and antioxidant activity and have been detailed and summarized elsewhere (39). In cell culture experiments, three critical factors should be kept in mind: the compound stability (40), concentrations used and whether unconjugated compounds or physiologically abundant phase II conjugates are used for most meaningful results (41, 42).

Some of these concerns can be efficiently addressed with a modified “*ex vivo*” cell culture setting. Therefore, serum or plasma samples of volunteers who ingested dietary supplements are added to cell culture mediums. Besides the advantage of substituting fetal calf serum (43) the human serum/plasma samples contain all bioavailable constituents and metabolites at physiological concentrations. The effects observed with this assay system should approximate cellular *in vivo* events. This approach has been successfully established after intake of Pycnogenol^®^ (35).

The gold standard for pharmacodynamic evaluations are randomized, placebo-controlled double-blinded clinical trials with healthy volunteers or patients. This study setting is increasingly used for determination of effects of dietary supplements including pine bark preparations (44). In this context, it should be noted that the precise composition of the extracts has to be taken into consideration (8). Clinical effects after intake of Pycnogenol^®^ have been documented for various pathophysiologic states such as cardiovascular diseases or diabetes mellitus and have been previously reviewed (2, 45–47).

## 2 Characteristics of Pycnogenol^®^

Pycnogenol^®^ is prepared in a patented extraction procedure (2) and constitutes the most extensively studied pine bark extract (8).

### 2.1 Botanical source

The French maritime pine bark extract Pycnogenol^®^ is produced from the outer protective bark of the French *Pinus pinaster* Aiton, subspecies *atlantica*, which is particularly resistant against salt and frost. These pine trees grow in the coastal southwest France over 30–50 years yielding consistent raw material (2).

### 2.2 Composition

The purified aqueous extract is a reddish-brown fine powder which is soluble in water. It contains low molecular weight compounds such as the flavonoids (+)-catechin, (-)-epicatechin, (+)-taxifolin, the benzoic acids protocatechuic acid, vanillic acid, gallic acid, and the cinnamic acids ferulic acid, caffeic acid or p-coumaric acid. These phenolic acids are present as glucosides, glucose esters and as aglycons (2, 45). In Pycnogenol^®^, higher molecular weight procyanidins predominate. These biopolymers composed of catechin and epicatechin consist of 2–12 monomeric subunits. Procyanidin dimers of the B-type such as B_1_, B_3_, or B_7_ have been identified in the extract (2).

### 2.3 Specifications

Since the specific pine tree species and the extraction process significantly influence the phytochemical composition and bioactivity of the extract (6), meaningful research results require reproducible composition of the product. The French maritime pine bark extract Pycnogenol^®^ is standardized to contain 65–75% total procyanidins (2) and it complies with the specifications for “Maritime Pine extract” monographed in the United States Pharmacopeia (USP). The USP specifies methods and requirements for the identification, evaluation of extract purity (e.g., microbial count, pesticides, residual solvents, heavy metals) and content of procyanidins.

## 3 Administration

Typically, preparations of Pycnogenol^®^ are orally ingested in form of capsules or tablets. Pycnogenol^®^ can also be added to drinking solutions (48). Furthermore, a dermal application has been explored (49). Daily oral doses of 30–360 mg have been investigated in clinical studies (47).

## 4 Absorption: compound bioavailability

The absorption of dietary polyphenols from the intestine into systemic blood circulation is generally considered to be low (50) and it might be affected by other food components such as dietary fiber, lipids, proteins, digestible carbohydrates, or divalent minerals (51). Smaller compounds such as catechins, caffeic acid or taxifolin can be absorbed in the small intestine and are usually present in blood samples approximately 0.5–2 h after ingestion (50, 52, 53). Procyanidin oligomers and polymers reach the large intestine where they are subjected to microbial metabolism yielding e.g., phenylvalerolactones (see section “6.1 Pre-systemic metabolism: role of gut microbiota”). Occasionally, procyanidin dimers, but no trimers have been reported in plasma. Microbial metabolites of procyanidins are bioavailable and detectable in blood samples after roughly 8–10 h following ingestion (11, 54). To the best of our knowledge, human pharmacokinetic studies of a pine bark extract have been only reported for Pycnogenol^®^.

### 4.1 Absorption into plasma/serum and concentration profiles

In a first pharmacokinetic study, eleven healthy volunteers ingested 300 mg Pycnogenol^®^ as a single dose and five volunteers received multiple doses of 200 mg Pycnogenol^®^ over 5 days (32). Plasma samples were analyzed over 14 h after single dose intake and 4 h after the last intake in the multiple dose regime (Table 2). The results allowed for describing time courses of five known compounds, namely catechin, taxifolin, caffeic acid, ferulic acid and the gut microbial metabolite 5-(3′,4′-dihydroxyphenyl)-γ-valerolactone (see also section “6.1 Pre-systemic metabolism: role of gut microbiota”). Catechin, caffeic acid, and ferulic acid were detectable 30 min after intake of Pycnogenol^®^, taxifolin after 2 h. As expected, the microbial metabolite 5-(3′,4′-dihydroxyphenyl)-γ-valerolactone appeared later in plasma after 6 h.

**TABLE 2:** Concentrations of constituents and metabolites determined after single (S) or multiple (M) intake of Pycnogenol^®^ by human volunteers (32, 70) or patients (34).

Compound	Plasma/serum concentration [ng/ml] Mean ± SD	References	Synovial fluid concentration [ng/ml] Mean ± SD	Blood cell concentration [ng/ml] Mean ± SD	Reference	Saliva concentration* [ng/ml]	Reference
Catechin	107.22 ± 55.49 (S; D300)	(32, 34)	2.99 ± 0.43 (M; D200)	71.18 ± 27.34 (M; D200)	(34)	n.a.	(70)
	48.56 ± 14.90 (M; D200)						
	52.53 ± 18.40 (M; D200)						
Taxifolin	33.34 ± 12.54 (S; D300)		0.21 ± 0.03 (M; D200)	0.52 ± 0.23 (M; D200)		4.68	
	≤*10.0*^a^ (M; D200)						
	0.20 ± 0.12 (M; D200)						
Ferulic acid	14.78 ± 5.89 (S; D300)		4.29 ± 1.83 (M; D200)	1.86 ± 0.36 (M; D200)		≤*3.32*^a^	
	18.71 ± 3.68 (M; D200)						
	3.02 ± 0.39 (M; D200)						
Caffeic acid	16.67 ± 13.29 (S; D300)		10.32 ± 3.96 (M; D200)	n.d. (M; D200)		1.79	
	2.42 ± 1.47 (M; D200)						
	9.28 ± 0.51 (M; D200)						
5-(3′,4′-dihydroxyphenyl)-γ-valerolactone	4.11 ± 2.08 (S; D300)		0.62 ± 0.77 (M; D200)	0.19 ± 0.07 (M; D200)		1.20	
	3.01 ± 0.31 (M; D200)						
	0.54 ± 0.84 (M; D200)						
Gallic acid	n.a.		n.a.	n.a.		10.34	
p-Coumaric acid	n.a.		n.a.	n.a.		3.64	
Protocatechuic acid	n.a.		n.a.	n.a.		8.24	

*Pilot investigation with one participant.

^a^Concentrations were below the LLOQ. n.d., not detected; n.a., not analyzed; LLOQ, lower limit of quantification. Single or daily doses ranged from 200 to 300 mg Pycnogenol^®^ (D200, D300).

**FIGURE 2:**
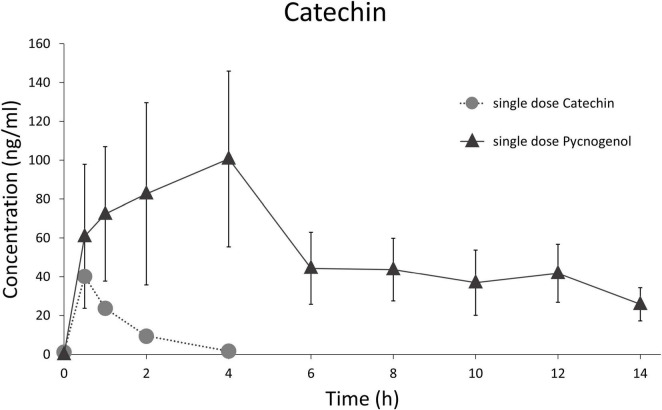
Comparison of time courses of total (free and conjugated) serum/plasma concentrations of catechin after administration of a single dose pure catechin (25 mg catechin, suspended in a matrix simulating a vegetable homogenate) (55) and a single dose of 300 mg Pycnogenol (32).

**FIGURE 3:**
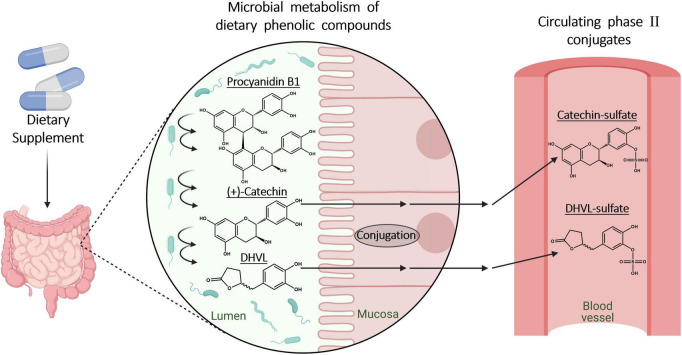
Microbial metabolic processes of dietary polyphenols and procyanidins in the gut and following phase II metabolism in enterocytes. Created with BioRender.com.

In this study, the plasma concentration profiles of catechin and ferulic acid were noteworthy (32). Catechin is a low molecular weight constituent of Pycnogenol^®^ and also present as biopolymer component in the procyanidin fraction. In the kinetic study, catechin concentration did not decline back to baseline levels after the peak concentration as it has been seen after ingestion of pure catechin (Figure 2) (55). After intake of Pycnogenol^®^, the plasma concentrations of catechin remained invariant between 6 and 12 h, and minimally declined after 14 h. This is suggestive of a prolonged formation of catechin via gut microbial degradation of procyanidins in the colon (Figure 3). Therefore, this procyanidin decomposition can be only observed with a delay of several hours, monomeric units such as epicatechin have not been determined in plasma 2 h after intake of procyanidins (56). The plasma concentrations of ferulic acid were also almost constant between 2 and 14 h after intake of Pycnogenol^®^ and the steady state concentrations after multiple doses appeared high compared to the single dose (32). This is again consistent with an ongoing production of ferulic acid, e.g., by methylation of caffeic acid, which in turn displayed low steady state concentrations. The examples of plasma concentrations of catechin and ferulic acid adumbrate the complex pharmacokinetics of dietary polyphenols.

In this pioneer pharmacokinetic study with Pycnogenol^®^, ten unknown compounds were found in plasma samples in addition to the identified ones (32). The plasma concentration profiles of the unknown molecules allowed for the prediction that six of the compounds were most likely constituents of the extract, while four compounds presumably represented gut microbial metabolites. Six of the unknown compounds were not detectable in steady state samples after multiple doses of the French maritime pine bark extract. Two of the molecules which were also determined under steady state conditions underwent phase II conjugation to a certain degree. Five of the compounds had a biphasic plasma concentration profile suggestive of, e.g., subjection to an enterohepatic circulation (see section “7 Elimination”). Unfortunately, these unknown constituents and metabolites could not be identified with the analytical equipment available at that time. In a later study, using quadrupole time of flight mass spectrometry (qTOF MS; see section “6.2 Systemic metabolism: conjugation and deconjugation”), eleven compounds, determined in serum samples of volunteers after daily intake of 200 mg Pycnogenol^®^, were assigned a molecular structure (33). Among those were taxifolin, cinnamic acids such as ferulic acid, phenylvaleric acid metabolites, as well as benzoic acids such as protocatechuic acid.

In a study with osteoarthritis patients (see section “5.2 Distribution into blood cells”), steady state serum concentrations were determined after intake of 200 mg Pycnogenol^®^ per day (34). Total concentrations of constituents and metabolites quantified in serum were highest for catechin, followed by caffeic acid, ferulic acid, 5-(3′,4′-dihydroxyphenyl)-γ-valerolactone, and taxifolin (Table 2). Compared to serum, higher concentrations of individual compounds were observed in other specimens, such as blood cells, endothelial cells, synovial fluid and saliva, indicating pronounced distribution processes *in vivo* (see section “5 Biodistribution”).

### 4.2 Dermal uptake

The transdermal bioavailability has been determined in an *in vitro* model system with human skin using a liquid preparation containing 5% Pycnogenol^®^. Within 12 h of perfusion, various smaller polyphenols were rapidly absorbed, e.g., catechin, taxifolin, protocatechuic acid (49). This suggests that Pycnogenol^®^ is suitable for dermal preparations as well.

## 5 Biodistribution

**FIGURE 4:**
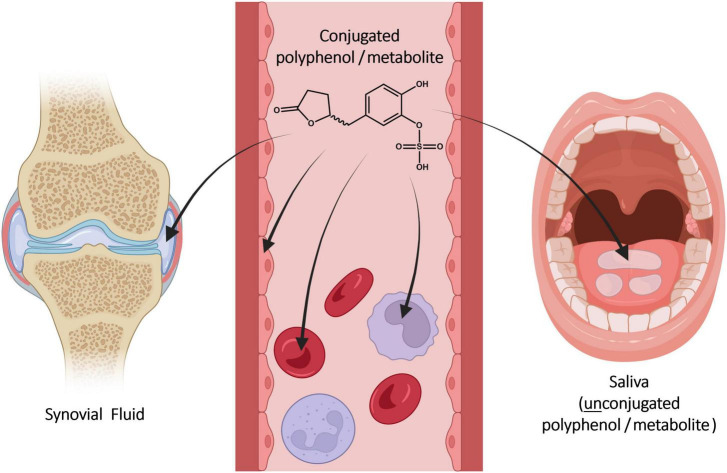
Distribution of constituents and metabolites from serum into blood cells, endothelial cells, synovial fluid, and saliva after ingestion of Pycnogenol. Created with BioRender.com.

Compound concentrations are typically determined in serum or plasma, although the systemic circulation is not necessarily the pharmacodynamic target compartment. It is assumed that bioactive compounds can reach target cells and tissues once they circulate in blood (Figure 4). The distribution of individual compounds and metabolites is relevant for pharmacokinetic and pharmacodynamic aspects.

### 5.1 Plasma protein binding

The binding to plasma proteins depends on the physicochemical characteristics of a compound and is typical for a given molecule (57). The extent of protein binding of bioactive molecules might impact pharmacokinetic and pharmacodynamic features (58). This substance characteristic was evaluated for various constituents and metabolites of Pycnogenol^®^ (23). The compounds’ binding to human serum albumin ranged from close to 100% for (+)-catechin and taxifolin to approximately 20% for protocatechuic acid (Table 3). The binding of cinnamic acids such as ferulic acid or caffeic acid was higher compared to the binding of benzoic acids like vanillic acid or gallic acid. The gut microbial metabolites 5-(3′,4′-dihydroxyphenyl)-γ-valerolactone and 5-(3′-methoxy-4′-hydroxyphenyl)-γ-valerolactone revealed with approximately 30% a rather low binding to albumin. It has to be critically mentioned that exclusively the protein binding of unconjugated polyphenols was determined in that study since conjugated reference compounds were not available. The degree of phase II conjugation, typically encompassing sulfation, glucuronidation and methylation (see section “6.1 Pre-systemic metabolism: role of gut microbiota”), is high for individual molecules (Table 3). More hydrophilic conjugates would be expected to display a decreased binding to plasma proteins, but published investigations suggested that a change in plasma protein binding was dependent on the polyphenol and its type and position of conjugation (59–62).

**TABLE 3:** Degree of phase II conjugation of compounds detected in serum/plasma after intake of Pycnogenol^®^ and plasma protein binding of Pycnogenol^®^ constituents and metabolites.

Compound	Conjugation degree [%] Mean ± SD	References	Plasma protein binding [%] Mean ± SD	Reference
Catechin	54.29 ± 26.77	(32, 34)	100	(23)
	56.50 ± 27.90			
Taxifolin	96.75 ± 7.23		100	
	100*			
Ferulic acid	90.32 ± 16.58		73.5 ± 0.12	
	100*			
Caffeic acid	80.95 ± 17.95		66.0 ± 0.23	
	69.40 ± 11.80			
5-(3′,4′-dihydroxyphenyl)-γ-valerolactone	98.34 ± 4.40		34.9 ± 1.28	
	100*			
5-(3′- methoxy-,4′-hydroxyphenyl)-γ-valerolactone	n.a.		26.4 ± 0.03	
Gallic acid	n.a.		31.6 ± 0.56	
p-Coumaric acid	n.a.		65.4 ± 4.84	
Protocatechuic acid	n.a.		20.7 ± 0.09	
Vanillic acid	n.a.		56.3 ± 1.16	
p-Hydroxy benzoic acid	n.a.		35.3 ± 10.9	
Procyanidin B1	n.a.		81.5 ± 1.09	

*A conjugation degree of 100% was assumed because no free concentrations were detectable. n.a.: not analyzed.

### 5.2 Distribution into blood cells

When determining blood concentrations of bioactive molecules, blood cells are usually disregarded and discarded after centrifugation to simplify analytical methods. However, these cells might represent an important compartment for disposition of active molecules though they have received less attention compared to plasma protein binding. The largest population of blood cells are erythrocytes which take up 99% of the cellular space of blood in humans. Pharmacologically active compounds might bind to the membrane or be taken up into red blood cells, where they can undergo metabolism (63). Red blood cells have also been reported to bind polyphenols such as gallic acid or resveratrol (64).

The binding of Pycnogenol^®^ constituents and the bioactive microbial metabolite 5-(3′,4′-dihydroxyphenyl)-γ-valerolactone to human erythrocytes was determined in an *in vitro* setting (19). Caffeic acid, ferulic acid and taxifolin showed binding to red blood cells while 5-(3′,4′-dihydroxyphenyl)-γ-valerolactone revealed accumulation within the cells. This conclusion was based on the observations that the erythrocyte / plasma partitioning ratio for 5-(3′,4′-dihydroxyphenyl)-γ-valerolactone exceeded tenfold that ratio of the other compounds, and that the uptake was decreased by the glucose transporter inhibitor phloretin and glucose. It was concluded that the metabolite undergoes facilitated uptake into red blood cells, possibly via GLUT-1 transporters. The detection of a glutathione adduct in the cells upon incubation with 5-(3′,4′-dihydroxyphenyl)-γ-valerolactone further confirmed its intracellular uptake as opposed to a mere binding to the cell membrane (19). In a similar way, binding and facilitated uptake of 5-(3′,4′-dihydroxyphenyl)-γ-valerolactone into human monocytes and a macrophage cell line were demonstrated (20).

In a clinical study with 33 patients suffering from severe osteoarthritis 16 patients were allocated to receive 200 mg Pycnogenol^®^ per day over 3 weeks before a scheduled knee arthroplasty surgery (34). Total concentrations of Pycnogenol^®^ constituents and metabolites were quantified in serum (see section “4.1 Absorption into plasma/serum and concentration profiles”), synovial fluid (see section “5.4 Distribution into synovial fluid”) and blood cells (Table 2). After 3 weeks of supplementation with Pycnogenol^®^, highest concentrations in blood cells were determined for catechin, ferulic acid, 5-(3′,4′-dihydroxyphenyl)-γ-valerolactone and taxifolin. Notably, the analyzed individual polyphenols displayed a preferential distribution between the investigated specimen with catechin and taxifolin being primarily present in blood cells compared to serum. Despite the previously observed facilitated uptake of 5-(3′,4′-dihydroxyphenyl)-γ-valerolactone into blood cells (19, 20), the concentrations of this metabolite were lower than expected in this patient cohort. This was explained by an ongoing further metabolism of 5-(3′,4′-dihydroxyphenyl)-γ-valerolactone in blood cells (see section “6.3 Site-specific metabolism: blood cells”) and confirmed by the detection of valerolactone-derived metabolites (34).

### 5.3 Distribution into endothelial cells

The vascular endothelium plays an essential role in cardiovascular health (65) and binding and/or uptake of dietary polyphenols to endothelial cells presumably contributes to beneficial health effects. Since Pycnogenol^®^ constituents and metabolites bound to blood cells, distribution into endothelial cells was additionally investigated (20). In an *in vitro* setting, the binding of the microbial metabolite 5-(3′,4′-dihydroxyphenyl)-γ-valerolactone to cells of the endothelial cell line EA.hy 926 was determined. As seen with blood cells, a fast high-capacity binding, which was diminished by phloretin, was recorded, suggesting that this metabolite is actively distributed into endothelial cells as well.

### 5.4 Distribution into synovial fluid

Local concentrations of bioactive molecules in joints are regarded important for the management of joint-related symptoms such as inflammation and pain. The highly permeable fenestrae of the synovial capillaries allow for a bidirectional exchange of small molecules between blood and synovial fluid. From the synovial microvessels, small solutes diffuse through the extracellular matrix of the synovial intima to reach the intraarticular space (66, 67). After intake of Pycnogenol^®^, constituents and metabolites were detected in synovial fluid samples of osteoarthritis patients undergoing knee arthroplasty (see section “5.2 Distribution into blood cells”) (34). Highest concentrations were determined for caffeic acid, besides ferulic acid, catechin, 5-(3′,4′-dihydroxyphenyl)-γ-valerolactone, and taxifolin (Table 2). As observed with blood cells and serum before, individual compounds showed a preferential distribution into synovial fluid compared to serum, namely 5-(3′,4′-dihydroxyphenyl)-γ-valerolactone, ferulic and caffeic acid.

### 5.5 Distribution into saliva

Saliva is a biofluid suitable for determination of drug concentrations as an alternative or addition to blood sampling via venipuncture. Typically, it is considered to be an ultrafiltrate of serum containing the free, non-protein-bound fraction of an active molecule (68, 69). Until recently, it was not known whether dietary polyphenols circulating in blood would be transferred into saliva. After developing an analytical method to quantify polyphenol concentrations in saliva, a saliva sample and a simultaneously obtained serum sample of a single volunteer were analyzed after intake of Pycnogenol^®^ (70). Gallic acid, protocatechuic acid, taxifolin, p-coumaric acid, caffeic acid and 5-(3′,4′-dihydroxyphenyl)-γ-valerolactone were detected in saliva. The concentrations of gallic acid, p-coumaric acid, taxifolin, and 5-(3′,4′-dihydroxyphenyl)-γ-valerolactone were higher in saliva as compared to serum (Table 2). In contrast, concentrations of protocatechuic acid, ferulic acid, and caffeic acid were higher in serum than in saliva. Although these results have to be confirmed with a higher number of participants, they might again suggest a preferential distribution of individual polyphenols. Notably, all compounds quantified in saliva were present in unconjugated form, presumably as a result of deconjugation by a β-glucuronidase (71) and/or arylsulfatase A (72) activity.

## 6 Metabolism

Dietary polyphenols and particularly procyanidins, which are highly prevalent in Pycnogenol^®^, undergo extensive *in vivo* metabolism. Some authors designate degradation of procyanidins by gut bacteria as “catabolism” and the resulting metabolites as “catabolites” to differentiate microbial from human metabolic processes. Gut microbial metabolic processes of dietary polyphenols and procyanidins have been detailed in various reviews (27, 41, 53, 54, 73–76), so only selected aspects will be delineated here.

### 6.1 Pre-systemic metabolism: role of gut microbiota

Although some oligomeric or polymeric procyanidins might be already subjected to microbial metabolism in the small intestine (77), up to 90% of ingested procyanidins have been estimated to reach the colon intact (78). In the large intestine, procyanidins and colonic microbiota are subjected to reciprocal interactions. On the one hand, the presence of oligomeric and polymeric procyanidins facilitates the proliferation of diverse bacteria with health-promoting effects for the human hosts, such as *Akkermansia muciniphila*, and butyrate-producing bacteria while inhibiting LPS-producing bacteria (54, 74). On the other hand, the procyanidins encounter a multitude of enzymatic reaction capabilities provided by bacterial strains, subjecting them to degradational bioconversion processes (74). In this context, simple hydrolysis of glycosides or glucuronides can occur as well as complex multi-step reactions converting procyanidins to phenyl-γ-valerolactones and phenylvaleric acids (41, 76). The procyanidins, composed of catechin and epicatechin, are disaggregated to monomeric units, which then undergo ring opening and fission reactions yielding valerolactones (Figure 3). While the formation of 5-(3′,4′-dihydroxyphenyl)-γ-valerolactone from (+)-catechin by intestinal bacteria has already been described by Das (79), only more recently the contributing colonic microbiota have been characterized. Both catechin and epicatechin can undergo bioconversion yielding, e.g., 5-(3′,4′-dihydroxyphenyl)-γ-valerolactone, by the metabolic reactions of *Eggerthelia lenta* and *Adlercreutzia equolifaciens* species, followed by activities of *Flavonifractor plautii* strains (80, 81). These gut microbial processes significantly contribute to generation of bioavailable metabolites from procyanidins (82). Besides the presence of certain microbial species in the colon, the yield of catechin and epicatechin monomers and their subsequent phenylvalerolactone metabolites also depends on the precise composition of the procyanidins. The B-type procyanidins, e.g., procyanidin B_2_, with monomers being linked via a single interflavan bond, are more readily cleaved compared to A-type procyanidins with two interflavan bonds (76).

After intake of Pycnogenol^®^, the microbial metabolites 5-(3′,4′-dihydroxyphenyl)-γ-valerolactone (previously also named δ-(3,4-dihydroxyphenyl)-γ-valerolactone or M1) and 5-(3′- methoxy-,4′-hydroxyphenyl)-γ-valerolactone (previously also called δ-(3- methoxy-,4-hydroxyphenyl)-γ-valerolactone or M2) were identified in urine samples of a volunteer as phase II conjugates (30). The formation of these valerolactones from procyanidins was confirmed by subsequent ingestion of the isolated procyanidin fraction of Pycnogenol^®^. This confirmed the metabolism of procyanidins and bioavailability of the valerolactone metabolites. Subsequently, the formation and bioavailability of 5-(3′,4′-dihydroxyphenyl)-γ-valerolactone after intake of Pycnogenol^®^ has been demonstrated for human plasma (32), serum (34), blood cells (34), synovial fluid (34), and saliva (70). In addition to the valerolactone, 4-hydroxy-5-(3′,4′-dihydroxyphenyl)-valeric acid and 4-hydroxy-5-phenyl-valeric acid were detected as well in serum samples after intake of Pycnogenol^®^ (33).

### 6.2 Systemic metabolism: conjugation and deconjugation

Dietary polyphenols and microbial metabolites of procyanidins typically undergo a phase II metabolism in enterocytes and liver cells to form glucuronic acid, sulfate, and methyl conjugates (Figure 3) (27, 41, 73, 82, 83). Due to the lack of commercially available reference compounds, the total concentrations of polyphenols and microbial metabolites are often measured after enzymatic cleavage of glucuronides and sulfates (11).

After intake of Pycnogenol^®^, phase II conjugates of constituents and metabolites were present in plasma / serum samples (32, 34). The samples were analyzed with and without enzymatic hydrolysis, revealing that the degree of conjugation varied among compounds. The lowest degree of conjugation showed catechin with approximately 55% (Table 3), followed by caffeic acid with 80%. An almost complete phase II conjugation with >90% was determined for ferulic acid, taxifolin and 5-(3′,4′-dihydroxyphenyl)-γ-valerolactone. The degree of conjugation was not measured for compounds in blood cells and synovial fluid due to insufficient sample volumes. In those cases, total concentrations after enzymatic incubation were reported (34). The enzymatic pretreatment of samples typically cleaves both glucuronides and sulfates so that it is not revealed which conjugate is the predominant one. The identity of phase II conjugates was to be uncovered in a pharmacokinetic study with 15 volunteers who received 200 mg Pycnogenol^®^ over 4 days. Serum samples were analyzed with quadrupole time of flight mass spectrometry (qTOF MS). In this study, exclusively sulfate conjugates of constituents and metabolites were identified (33). Whether a phase II conjugation entails sulfation, glucuronidation and/or methylation primarily depends on the substrate properties and the ingested dose. The administration of lower doses favors sulfation since the involved enzymes have a high substrate affinity, but lower capacity compared to glucuronidation processes (84).

Compared to the unconjugated structures, phase II conjugates have altered physicochemical properties; they are more polar and cannot readily enter cells by diffusion. The bioactivity of the conjugates is generally reported to be significantly attenuated (41, 42, 85). The apparent contradiction that *in vivo* activity is observed despite the fact that most of the circulating polyphenolic compounds are phase II conjugates, has been explained by a local “deconjugation in inflammation” (86). On a cellular level, conjugates have been shown to be subjected to deconjugation by enzymes such as glucuronidases bound to the cell surface. The deconjugation was enhanced under inflammatory conditions (86–88). It has been suggested that phase II conjugation stabilizes the molecule and that conjugates represent carriers for the transport in circulation (42, 89, 90).

After intake of Pycnogenol^®^, a compartment-specific deconjugation of phase II metabolites has been observed in a human volunteer (see section “5.5 Distribution into saliva”). In saliva, but not in serum, various polyphenols, and the microbial metabolite 5-(3′,4′-dihydroxyphenyl)-γ-valerolactone were exclusively present as unconjugated molecules (70). This might be the first report of metabolite deconjugation in humans.

### 6.3 Site-specific metabolism: blood cells

The observation of a facilitated uptake of 5-(3′,4′-dihydroxyphenyl)-γ-valerolactone into blood cells and its determination in cell samples of osteoarthritis patients after intake of Pycnogenol^®^ (see section “5.2 Distribution into blood cells”) prompted systematic advances to elucidate the detailed metabolism of 5-(3′,4′-dihydroxyphenyl)-γ-valerolactone in human blood cells (28). Metabolite profiling using a metabolomic approach revealed the generation of six confirmed and seven putative metabolites. Among the identified compounds were two glutathione conjugate isomers, conjugates with cysteine, sulfate and oxidized glutathione and other biotransformation products such as phenylvaleric acid derivatives. Notably, six of those metabolites were subsequently also detected in blood cell samples of human volunteers after ingestion of multiple doses of 200 and 300 mg Pycnogenol^®^ (28). This indicated that human blood cells are active sites of metabolism yielding structurally diverse biotransformation products.

## 7 Elimination

The major excretion routes of dietary polyphenols and their metabolites include renal and biliary elimination of compounds circulating in blood. It was suggested that smaller conjugates are more likely to undergo renal elimination. Compounds that undergo conjugation in the liver and larger conjugates are presumably exported into the bile and then back into the small intestine where they might be reabsorbed (42, 73). Suggestive for such an enterohepatic circulation is a second, lower plasma peak concentration some hours apart from the first peak (91).

An excretion with feces is another route of elimination for dietary polyphenols or their metabolites (27, 54). This represents a minor pathway since only small amounts of phenolic compounds or metabolites have been recovered from fecal samples (11, 91). Compounds may undergo fecal elimination if they were not absorbed in the intestine or were excreted from enterocytes back into the intestinal lumen via efflux transporters (73). Likewise, metabolites undergoing biliary elimination might escape reabsorption and may be found in fecal samples. Such a transfer back into the gut lumen would be consistent with the recovery of labeled resveratrol in feces of human volunteers after an intravenous dose (91).

Renal elimination of constituents and metabolites of the French maritime pine bark extract Pycnogenol^®^ has been reported for human volunteers (30, 31). After intake of 100 or 200 mg Pycnogenol^®^, free as well as sulfate and glucuronide conjugates of ferulic acid were determined in urine samples. Ferulic acid was suggested as marker of consumption of French maritime pine bark extract (31). After consumption of 5.28 and 1.06 g Pycnogenol^®^ by one volunteer, conjugates of ferulic acid and taxifolin were found in urine samples 2–3 h after intake. After 8–12 h, the microbial metabolites 5-(3′,4′-dihydroxyphenyl)-γ-valerolactone and 5-(3′-methoxy-4′-hydroxyphenyl)-γ-valerolactone (see section “6.1 Pre-systemic metabolism: role of gut microbiota”) were present in urine (30).

As for the half-lives (t½) of Pycnogenol^®^ components and metabolites *in vivo*, small molecules revealed short to intermediate terminal t½ of 4.42 ± 2.47 h for caffeic acid, 6.87 ± 3.83 h for ferulic acid and 8.89 ± 2.81 h for taxifolin (32). Catechin as a single compound has a rather short half-life. In the presence of high molecular weight procyanidins, however, it is apparently continuously produced by the activity of gut microbiota releasing catechin and epicatechin units from procyanidin oligomers and polymers (Figure 2) (32, 55). This also applies for the catechin-derived microbial metabolite 5-(3′,4′-dihydroxyphenyl)-γ-valerolactone, which was detectable in blood at least over 14 h (32). The t½ of 5-(3′,4′-dihydroxyphenyl)-γ-valerolactone might be short, but there is an extended production and absorption, presumably as long as the procyanidins are present in the gut (46). However, since pharmacokinetic studies are complex and therefore rare, this issue has not been investigated in more detail yet.

## 8 Summary

The proprietary product Pycnogenol^®^ originates from a definite French maritime pine species and has been researched in a multitude of human studies. This extract is standardized to contain 65–75% procyanidins which are biopolymers of catechin and epicatechin units. The basis for all clinical effects is the absorption, distribution, metabolism and elimination of extract constituents and metabolites. To the best of our knowledge, human pharmacokinetic studies investigating these aspects in detail in a procyanidin-rich pine bark extract, have been only performed for Pycnogenol^®^.

Low molecular weight constituents, such as catechin, caffeic and ferulic acid, taxifolin are readily absorbed from the small intestine and typically detectable in blood after 30 min. There are indications for a prolonged presence of catechin and ferulic acid in the systemic circulation. This is presumably due to a continuous generation of catechin from the degradation of procyanidins by gut microbiota and formation of ferulic acid by methylation of caffeic acid. In the lower intestine, microbial strains have been reported, that are involved in the breakdown of procyanidins into catechin and epicatechin monomers which are thereafter transformed into phenylvalerolactones such as 5-(3′,4′-dihydroxyphenyl)-γ-valerolactone. The phenylvalerolactones are bioavailable compounds which appear in blood samples approximately 8 h after intake of the extract. Compounds present in the blood typically undergo an extensive phase II metabolism in the enterocytes and hepatocytes yielding sulfate and glucuronic acid conjugates. After intake of Pycnogenol^®^, a high degree of conjugation (>90%) was seen for taxifolin, ferulic acid and 5-(3′,4′-dihydroxyphenyl)-γ-valerolactone. In contrast, the degree of conjugation was lower for catechin (≈ 55%) and caffeic acid (≈ 80%). Only sulfate conjugates were observed in one study.

Constituents and metabolites originating from the extract are not solely present in plasma or serum, they reveal an individual distribution in blood cells and synovial fluid as well. Thereby, catechin and taxifolin were mainly present in blood cells while 5-(3′,4′-dihydroxyphenyl)-γ-valerolactone, ferulic acid and caffeic acid preferentially resided in synovial fluid of osteoarthritis patients. Additional *in vitro* experiments suggested a facilitated uptake of 5-(3′,4′-dihydroxyphenyl)-γ-valerolactone into erythrocytes, monocytes and endothelial cells, probably via GLUT-1 transporters. After intake of Pycnogenol^®^, gallic acid, protocatechuic acid, taxifolin, p-coumaric acid, caffeic and 5-(3′,4′-dihydroxyphenyl)-γ-valerolactone were also detected in saliva. Notably, the compounds were exclusively present in their deconjugated form and the comparison with a simultaneously obtained serum sample suggested a preferential distribution between serum and saliva which needs to be confirmed in a bigger study.

The main route of elimination of constituents and metabolites of the French pine bark extract is the renal excretion. As a metabolite that was only determined in urinary, but not in blood samples, 5-(3′-methoxy-4′-hydroxyphenyl)-γ-valerolactone was identified.

The accumulated broad knowledge regarding the pharmacokinetics of compounds and metabolites of Pycnogenol^®^ constitute a rational basis for effects characterized on a cellular level and observed in human clinical studies.

## Author contributions

JB: Conceptualization, Visualization, Writing – original draft, Writing – review and editing. PH: Conceptualization, Supervision, Writing – original draft, Writing – review and editing.
